# DEL-Thyroid: deep ensemble learning framework for detection of thyroid cancer progression through genomic mutation

**DOI:** 10.1186/s12911-024-02604-1

**Published:** 2024-07-22

**Authors:** Asghar Ali Shah, Ali Daud, Amal Bukhari, Bader Alshemaimri, Muhammad Ahsan, Rehmana Younis

**Affiliations:** 1 Center of Excellence in Artificial Intelligence (CoE-AI), Department of Computer Science, Bahria University, Islamabad, 04408 Pakistan; 2 Faculty of Resilience, Rabdan Academy, Abu Dhabi, United Arab Emirates; 3 Department of Information Systems and Technology, Collage of Computer Science and Engineering, University of Jeddah, Jeddah, Saudi Arabia; 4 Software Engineering Department, College of Computing and Information Sciences, King Saud University, Riyadh, Saudi Arabia; 5 Department of Computer Science, University of Alabama at Birmingham, 1402 10th Avenue S, Birmingham, AL 35294 USA; 6 College of Letters and Sciences, Graduate Student of Robotics Engineering, Columbus State University, Columbus, USA

**Keywords:** Thyroid Cancer, Deep learning, Long short-term memory (LSTM), Ensemble learning model (ELM), Bi-directional LSTM (Bi-LSTM), Gated recurrent units (GRUs), Mutation detection

## Abstract

Genes, expressed as sequences of nucleotides, are susceptible to mutations, some of which can lead to cancer. Machine learning and deep learning methods have emerged as vital tools in identifying mutations associated with cancer. Thyroid cancer ranks as the 5th most prevalent cancer in the USA, with thousands diagnosed annually. This paper presents an ensemble learning model leveraging deep learning techniques such as Long Short-Term Memory (LSTM), Gated Recurrent Units (GRUs), and Bi-directional LSTM (Bi-LSTM) to detect thyroid cancer mutations early. The model is trained on a dataset sourced from asia.ensembl.org and IntOGen.org, consisting of 633 samples with 969 mutations across 41 genes, collected from individuals of various demographics. Feature extraction encompasses techniques including Hahn moments, central moments, raw moments, and various matrix-based methods. Evaluation employs three testing methods: self-consistency test (SCT), independent set test (IST), and 10-fold cross-validation test (10-FCVT). The proposed ensemble learning model demonstrates promising performance, achieving 96% accuracy in the independent set test (IST). Statistical measures such as training accuracy, testing accuracy, recall, sensitivity, specificity, Mathew's Correlation Coefficient (MCC), loss, training accuracy, F1 Score, and Cohen's kappa are utilized for comprehensive evaluation.

## Introduction

The identification of cancer in 1786 by Caleb Parry marked the initiation of extensive research into its various types and etiologies. Thyroid cancer ranks as the fifth most prevalent cancer among both men and women in the USA [1]. It manifests primarily in two forms based on hormone production: hypothyroidism and hyperthyroidism [2]. Hypothyroidism occurs when the thyroid gland produces an excess of thyroid hormone, while hyperthyroidism arises from insufficient production. Thyroid hormone plays a crucial role in human metabolism. The spectrum of thyroid cancer includes follicular thyroid cancer, papillary thyroid cancer, anaplastic thyroid cancer, hurtles cell cancer, anaplastic thyroid cancer, and medullary thyroid cancer (MTC). Symptoms such as neck lumps, throat pain, difficulty swallowing, coughing, and hoarseness are indicative of thyroid cancer.

Mutation is one of the primary causes of thyroid cancer. Any alteration to the gene sequence is referred to as a mutation [3]. This research aims to provide a computational framework for identifying the mutations that lead to thyroid cancer.

Researchers have already presented several computational studies for the identification of thyroid cancer. Some of the most recent techniques are thoroughly addressed in this area of the research. Researchers employed the texture they presented to identify thyroid nodules in various thyroid cancer situations. This work uses texture analysis and mathematical models to describe visual inhomogeneity. The CAD and texture analysis are done using the PubMed/MEDLINE database. The study concludes that a better way to categorize thyroid nodules in cases of thyroid cancer is through the texture analysis of photos using machine learning and deep learning methodologies. Examples of numerous investigations using machine learning-based texture analysis are given in the report. In US Texture Analysis of thyroid nodules, the results demonstrate an accuracy of 90% for computerized B-mode texture analysis, 96% with SVM, and 90.9% for random texture features [4]. [5] This study explores the efficacy of a radiomics model based on CT imaging for distinguishing between thyroid cancer. By analyzing 376 cases and employing advanced feature selection techniques, the model achieved a high diagnostic accuracy 99.13%. but this study is on CT imaging. The three MLP models employed are MLP-1, MLP-2, and MLP-3. With an output layer, MLP-1 uses seven independent factors as input, including gender, age, location of nodal disease, tumor size, race, and number of positive lymphocytes [6].

The tall cell subtype (TC-PTC) of papillary thyroid carcinoma (PTC) is notably aggressive, characterized by its difficult-to-maintain definition, leading to high inter-observer variability. A multicenter study validated a deep learning (DL) algorithm for detecting tall cells in 160 externally collected HE-stained PTC whole-slide images, achieving a sensitivity of 90.6% and specificity of 88.5% for TC detection. The algorithm’s accuracy in detecting non-TC areas was also high, and its use correlated significantly with relapse-free survival, demonstrating robust performance without retraining [7]. A study [8] demonstrated that combining a convolutional neural network classifier with PRS significantly improved diagnostic accuracy, elevating the AUROC from 0.83 to 0.89 and achieving a sensitivity 95% and specificity 63%. This study obtained a thyroid prognostic accuracy of 94.5%. Both human and follicular thyroid cancer are recognized using Raman microscopic imaging [9]. The spectrum pre-processing is done in MATLAB. This study demonstrates FTC-133 Distinction accuracy of 88.9%.

The most recent study employs bioinformatics techniques to find thyroid cancer biomarkers. This investigation uses data from the Gene Expression Omnibus database (GEO). The following four datasets are combined for this investigation: GSE33630, GSE3467, GSE3678, and GSE53157. 64 samples of normal tissue and 100 samples of thyroid cancer, 164 samples were re-selected from the dataset. Using the Robust Rank Aggreg (RRA) approach, the differentially expressed genes (DEG) are discovered. On these GEOs, many procedures are conducted, including pathway analysis, survival analysis, gene ontology (GO) [10], functional annotation, and protein-protein interaction (PPI) analysis [11].

In another study, the six prognoses of papillary thyroid cancer are identified by multi-omics [12] and bioinformatics analysis. Additionally, this work employs the GEO database, which has 164 unregulated and 168 downregulated DEGs. These DEGs underwent KEG G and Go analyses to produce the PPI network and hub genes, which are then extracted [13]. In a study employing integrated bioinformatics analysis, W. Liang and F. Sun identified the important genes in papillary thyroid cancer [14]. The study uses four datasets—GSE3678, GSE3467, GSE33630, and GSE58545—and applies KEGG pathway analysis [15], Kyoto Encyclopaedia [16], and Gene ontology (GO) [10] to the development of PPI networks [17]. This study found 114 DEGs with downregulation and 111 with upregulation. According to the study, BCL2, CCND1, and COL1A1 genes may be the main cause of papillary thyroid cancer.

According to a recent study, AI and ML produce accurate thyroid nodule estimates [18]. This study demonstrates that AI technologies can identify thyroid nodules more accurately. Additionally, deep learning techniques effectively classify cancerous and benign thyroid cancers. In this study, 187 patients’ data are used for testing, and 592 patients’ data are used for training. In this work, a 10-FCVT is used with the VGG-16T model. The model’s accuracy reading is 86.43% [19]. ThyNet model is created using deep learning and AI models to distinguish between benign and malignant thyroid tumors, increasing the effectiveness of the radiography procedure. This model used an 8339 patients dataset containing 18,049 images and gave an accuracy of 92.2% [20]. Some similar work is also implemented on other deseases [21–26]. Table 1 explains the summary of the Literature review of the past researchers.

**Table 1:** Summary of the literature review

Paper Citation	Methodology	Results
[4]	Texture analysis with Machine learning	90% accuracy for computerized B-mode texture analysis 96% accuracy with SVM 90.9% with random transform features
[6]	Machine learning algorithms with Fisher’s discriminant ratio, Kruskal-Wallis’ analysis, and Relief-F on the SEER database	94.5% accuracy
[7]	Deep Learning Based Algorithms	Sensitivity 90.6% Specificity 88.5%
[8]	Deep Learning Model	AUC 89% Sensitivity 95% Specificity 63%
[9]	Raman Spectrograph	88.9% accuracy
[11]	Bioinformatics strategy on GEO database	7 key genes in the PPI network are the therapeutic targets of thyroid cancer
[13]	Multi-omics and bioinformatics analysis	OGN, FGF13CDH3, CTGF, CYR61, CHRDL1, are key genes for papillary thyroid cancer
[14]	Integrated Bioinformatics Analysis	CCND1, COL1A1, and BCL2 are the genes for papillary thyroid carcinoma
[19]	VGG-16T CNN model	86.43% Accuracy
[20]	ThyNet	92.2% Accuracy

The most recent studies provided to identify thyroid adenocarcinoma are discussed in a literature review. These studies do, however, have certain limitations as follows:

- A generalized dataset was not employed in most research.
- Most of the effort is focused on the patient ultrasound images from the hospital dataset.
- Only four to ten genes are often found in studies for thyroid cancer diagnosis.
- The models from the earlier study are not best evaluated using various statistical tools.
- None of the studies listed above used any ELM to identify thyroid adenocarcinomas.

The proposed model in this study is developed to overcome these limitations. The mutation information of 40 genes that cause thyroid cancer is derived from https://intogen.org/ [27] and the normal gene sequences are downloaded from https://asia.ensembl.org/ [28] with web scraping code written in Python. The contributions of this study are as follows:

- Constructed a benchmark mutated dataset by integrating mutation information into normal gene sequences and produced a generalized and updated carcinogenic mutation dataset crucial for novel studies.
- Developed composition-dependent and position-variant features for single-nucleotide, bi-nucleotide, and tri-nucleotide configurations feature extraction techniques extracting 522 features per carcinogenic mutation.
- Proposed Ensemble deep learning framework consist of multiple deep learning algorithms (LSTM, GRU, and BLSTM) enables the development of an early detection diagnostic system for thyroid cancer based on genomic data.
- Tested trained models on a test dataset and compared performances, achieving a high accuracy of 96%.
- Enhances personalized thyroid cancer detection and treatment for individual patients.

## Materials and methods

**Fig. 1:**
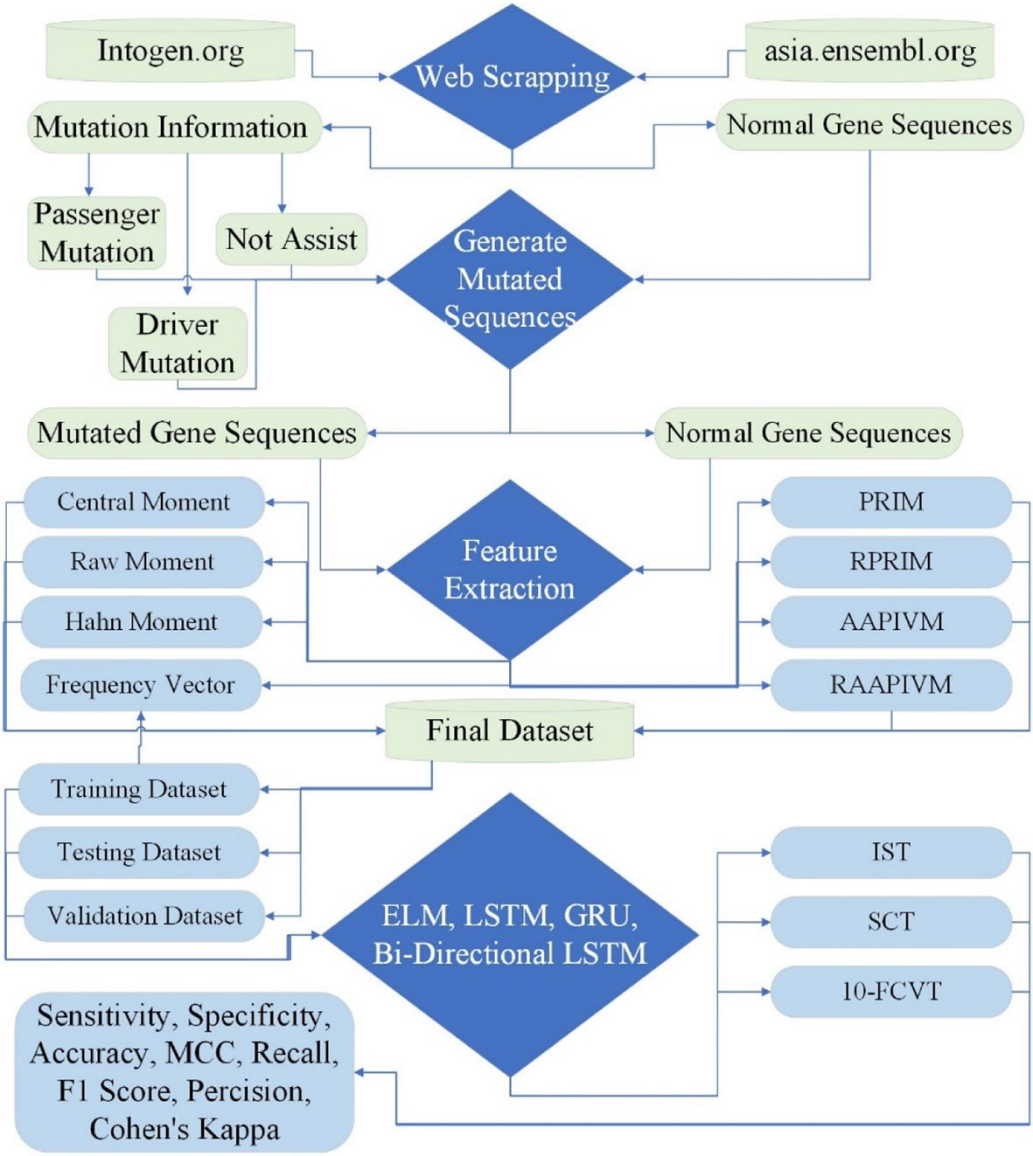
Research methodology for thyroid adenocarcinoma detection

The proposed study developed an ELM consisting of LSTM [29], GRU [30], and BLSTM [31] for the early detection of mutation in genes causing thyroid cancer. The proposed methodology of this study is explained in Fig. 1.

### Data collection

Data collection and pre-processing is one of the key factors for training a model. Before feeding the data to the model, the essentials are cleaning, pre-processing, and normalizing [32]. The pre-processed dataset gives efficient machine learning and deep learning model results.

There is no generalized dataset available for thyroid adenocarcinoma. So, the proposed study developed a complete process for data collection. The normal gene sequences for thyroid adenocarcinoma are extracted from asia.ensambl.org [28], and the mutated gene sequences for thyroid adenocarcinoma are extracted from intogen.org [27]. Web scrapping code [33] is written in Python to automatically extract the required normal gene sequences from a well-known database, asia.ensembl.org, and the associated mutation information from a well-known mutation database, intogen.org. Mutated sequences are obtained by applying mutation information on normal gene sequences. Driver mutation causes cancer [34, 35]; therefore, only driver mutations related to thyroid adenocarcinoma are considered while creating the mutated dataset.

There are 696 gene mutations involved in thyroid adenocarcinoma caused by 40 driver genes. All the samples are collected from persons of different ages, genders, treatments, cancer, and normal physical conditions. 696 mutations are used to train, test, and validate the model. Table 2 shows 40 genes related to thyroid adenocarcinoma, having 696 mutations of 633 samples in the dataset.

**Table 2:** Detail of the genes involved in thyroid adenocarcinoma

Symbol	Mutation	Sample	Symbol	Mutation	Sample
BRAF	371	371	ATM	9	6
NRAS	53	53	EIF1AX	9	6
KMT2C	34	26	RBFOX2	7	6
HRAS	23	22	KMT2A	8	6
NEFH	16	18	PRR14	7	6
PABPC1	17	12	PAK2	5	5
FAM186A	10	10	AKT1	6	5
HERC2	15	8	KRAS	7	4
DNMT3A	6	7	PDPR	5	4
TP53	8	7	WNK2	5	4
NF1	5	2	RET	6	4
CUX1	2	2	CHEK2	4	4
EPHA7	2	2	RHPN2	4	3
PDE4DIP	12	2	ARID2	2	3
PTEN	4	2	RGPD3	4	3
LRP1B	6	2	STAG2	2	2
MAP3K1	2	2	SETBP1	2	2
ABL2	3	2	PPP2R1A	2	2
FAT3	8	2	HSP90AA1	2	2
USP6	1	2	DGCR8	2	2

The benchmark dataset for this work is represented by Eq. (1)

1 $$\:T=\:{T\:}^{+}\:U\:{T}^{-}$$

In the Eq. (1) $\:T$ represents a balanced dataset, $\:{T}^{+}$ are the normal gene sequences and $\:{T}^{-}$ are the mutated gene sequence for thyroid adenocarcinoma. $\:U$ shows the union of these sequences to create an accurate, balanced dataset.

### Feature extraction

Feature extraction is the dimension reduction process by removing the redundant and irreverent data from the dataset and extracting the main data features to increase the accuracy, learning rate, and results of the machine learning without losing useful data information [36, 37]. It is one of the machine learning algorithm’s most important steps in data processing. For the proposed study, a sequential model is used to express the gene sequence in thyroid cell nucleotides represented by Eq. (2) [38].

2 $$\:{x}_{1}{x}_{2}{x}_{3}{x}_{4\:}{x}_{5}{x}_{6}\dots\:\dots\:\dots\:.{x}_{60}$$

In the equation $\:{X}_{1}$ represent the first gene in the thyroid cell sequence and $\:{X}_{60}$ represents the last gene of the sequence. 40 is the total number of genes involved in the Thyroid adenocarcinoma.

#### Statistical moment

Statistical moments is a quantitative analysis describing gene distribution in the gene sequences [39]. The proposed study uses statistical moments to convert genomic data into fixed sizes for utilization in ELM. Raw Moment [40], Hahn moment [41], and the central moment [4] are used in the proposed model for describing the gene data properties. Raw moment describes the position of the genes in the specific nucleotides. It is also used in the probability distribution of the genes in gene sequences. The central moment is location invariant and uses data centroids for calculations. The Hahn moment uses the Hahn polynomial to extract features from the gene sequences [42]. All these moments are used to find information regarding the positioning and the composition of the genes in the nucleotides. As the genes are in sequential manners, they use a 2-dimensional matrix. The 2D matrix of the gene resides inside the nucleotide, is described in Eq. (3).

3 $$\:{G}^{{\prime\:}}=\:\left[\begin{array}{ccc}{G}_{11}&\:{G}_{12}\dots\:.&\:{G}_{1 N}\\\:{G}_{\begin{array}{c}21\\\:.\\\:.\\\:.\\\:.\end{array}}&\:{G}_{\begin{array}{c}22\dots\:\dots\:.\\\:.\\\:.\\\:.\\\:.\end{array}}&\:{G}_{\begin{array}{c}2 N\\\:.\\\:.\\\:.\\\:.\end{array}}\\\:{G}_{N1}&\:{G}_{N2\:.\:\dots\:\dots\:}&\:{G}_{\begin{array}{c}NN\\\:\:\end{array}}\end{array}\right]$$

In the equation $\:{G}^{{\prime\:}}$ represents the 2D matrix of thyroid cancer genes and $\:{G}_{11}\:to\:{G}_{NN}$ represents the genes resides at specific locations inside this 2D matrix. The Raw moment $\:R(a,\:b)$ for 2D matrix $\:{G}^{{\prime\:}}\:$is calculated by Eq. (4)

4 $$\:{R}_{ab}=\:{\sum\:}_{p=1}^{N}{\sum\:}_{q=1}^{N}{p}^{a}{q}^{b}{G}^{{\prime\:}}\left(p,q\right)$$

In the equation $\:{R}_{ab}$ represents the raw moments at the degree of $\:a+b$, $\:{G}^{{\prime\:}}\left(p,q\right)$ is 2D matrix of the genes at any point $\:p$ and $\:q$. For the calculation of the central moment, the centroid of the gene is calculated, represented by Eq. (5)

5 $$\:{C}_{ab}=\:{\sum\:}_{p=1}^{N}{\sum\:}_{q=1}^{N}{(p\:-\:\stackrel{-}{x})}^{a}{(q\:-\stackrel{-}{\:y})}^{b}{G}^{{\prime\:}}\left(p,q\right)$$

In Eq. (4) $\:{C}_{ab}$ is the central moment, $\:\:\stackrel{-}{x}\:and\:\stackrel{-}{y}$ represents the centroids of the gene dataset. Hahn polynomial is calculated by Eq. (6)

6 $$\:{h}_{n}^{x,y\:}\left(r,N\right)={(N+V-1)}_{n}{\:(N-1)}_{n}\times\:\sum\:_{k=0}^{n}{(-1)}^{k}\frac{{(-n)}_{k}{(-r)}_{k\:}{(2 N+u+v-n-1)}_{k\:}}{{(N+v-1)}_{k\:}{(N-1)}_{k\:}}\:\frac{1}{k!}$$

The equation uses pochammer notation and gamma operators [43]. The Hahn moment calculated by the Hahn polynomial is illustrated in Eq. (7)

7 $$\:{H}_{pq}={\sum\:}_{p=0}^{N-1}{\sum\:}_{q=1}^{N-1}{{G}^{{\prime\:}}\left(p,q\right)\:h}_{n}^{\stackrel{-}{x,y}\:}\left(q,\:N\right){h}_{j}^{\stackrel{-}{x,y}\:}\left(p,\:N\right)$$

In the Eq. (7) $\:{H}_{pq}$ represents the Hahn moment using the Hahn polynomial.

#### Position relative incident Matrix (PRIM) and reverse position relative incident Matrix (RPRIM) calculation

Any gene is formed by the combination of nucleotides and their sequences. Any computational model is built by finding the positioning of the nucleotide in a gene. In the proposed study, it is very important to find the position of each nucleotide and its binding in the gene. PRIM [44] calculates the positioning of each nucleotide inside the gene sequence. Equation (8) illustrates the PRIM for the 10 by 10 matrix.

8 $${P}_{PRIM}= \left[\begin{array}{cccccc} {P}_{1 \to 1}& {P}_{1 \to 2}& \cdots & {P}_{1 \to j}& \cdots & {P}_{1 \to 20}\\ {P}_{2 \to 1} & {P}_{2 \to 2} & \cdots & {P}_{2 \to j } & \cdots & {P}_{2 \to 20}\\ \vdots & \vdots & & \vdots & & \vdots\\ {P}_{ n \to 1 } & {P}_{ n \to 2 } & \cdots & {P}_{ n \to j } & \cdots & {P}_{ n \to 20 }\\ \vdots & \vdots & & \vdots & & \vdots\\ {P}_{m \to 1 }& {P}_{m \to 2 }& \cdots & {P}_{m \to j }& \cdots & {P}_{m \to 20} \end{array}\right]$$

In Eq. (8) $\:P$ are the nucleotide at a specific position in the gene sequence, and a 10 by 10 matrix is used. RPRIM [45] is applied on the gene sequences the same way PRIM is applied but in the reverse sequence shown in Eq. (9).

9 $${P}_{RPRIM} = \left[\begin{array}{cccccc} {P}_{1 \to 1}& {P}_{1 \to 2}& \cdots & {P}_{1 \to j}& \cdots & {P}_{1 \to 20}\\ {P}_{2 \to 1} & {P}_{2 \to 2} & \cdots & {P}_{2 \to j } & \cdots & {P}_{2 \to 20}\\ \vdots & \vdots & & \vdots & & \vdots\\ {P}_{ n \to 1 } & {P}_{ n \to 2 } & \cdots & {P}_{ n \to j } & \cdots & {P}_{ n \to 20 }\\ \vdots & \vdots & & \vdots & & \vdots\\ {P}_{m \to 1 }& {P}_{m \to 2 }& \cdots & {P}_{m \to j }& \cdots & {P}_{m \to 20} \end{array}\right]$$

#### Feature vector determination

Frequency vector distribution is used to find the occurrence of every nucleotide in the gene sequence [46]. The gene distribution in the proposed study is calculated by Eq. 10.

10 $$\:\alpha\:=\left\{{\beta\:}_{1},\:{\beta\:}_{2},\:{\beta\:}_{3}\dots\:.\:{\beta\:}_{n}\right\}$$

The Eq. (9) $\:\alpha\:$ is the frequency distribution vector. $\:{\beta\:}_{1},\:{\beta\:}_{2},\:{\beta\:}_{3}$ are the overall count of specific elements of gene sequence.

#### Position incidence vector calculation

Determining the feature vector reveals the presence of nucleotides within a particular gene sequence. The Accumulative Absolute Position Incidence Vector (AAPIV) [47] consolidates the positional occurrences of these genes. Equation (11) quantifies the positional distribution of genes across nucleotides.

11 $$\:P=\{{\lambda\:}_{1},\:{\lambda\:}_{2},\:{\lambda\:}_{3},\:\dots\:\dots\:.{\lambda\:}_{N}\}$$

The $\:nth$ part is calculated by Eq. (12)

12 $$\:{\:\lambda\:}_{N}=\:{\sum\:}_{k=1}^{n}{\beta\:}_{k}$$

The reverse AAPIV is the same way as AAPIV but in reverse order of gene sequences. The equation for the calculation of Reverse AAPIV is represented by

13 $$\:{P}_{R}=\{{\lambda\:}_{1},\:{\lambda\:}_{2},\:{\lambda\:}_{3},\:\dots\:\dots\:.{\lambda\:}_{N}\}$$

In the equation $\:{P}_{R}$ represents the Reverse AAPIV, $\:{\lambda\:}_{1}\:to\:\:{\lambda\:}_{n}$shows the gene sequences from 1 to $\:n$.

### Algorithm for predictive modelling

The proposed study developed an ELM of LSTM, GRU, and BLSTM to identify thyroid adenocarcinoma. The details of LSTM, GRU, BLSTM, and ELM are explained in subsections.

#### Long short-term memory network (LSTM)

LSTM is used to remove the vanishing gradient problem. The information in LSTM passes through different gates. LSTM uses cells; the cell consists of three gates: forget gate, input gate, and output gate [48].

**Fig. 2:**
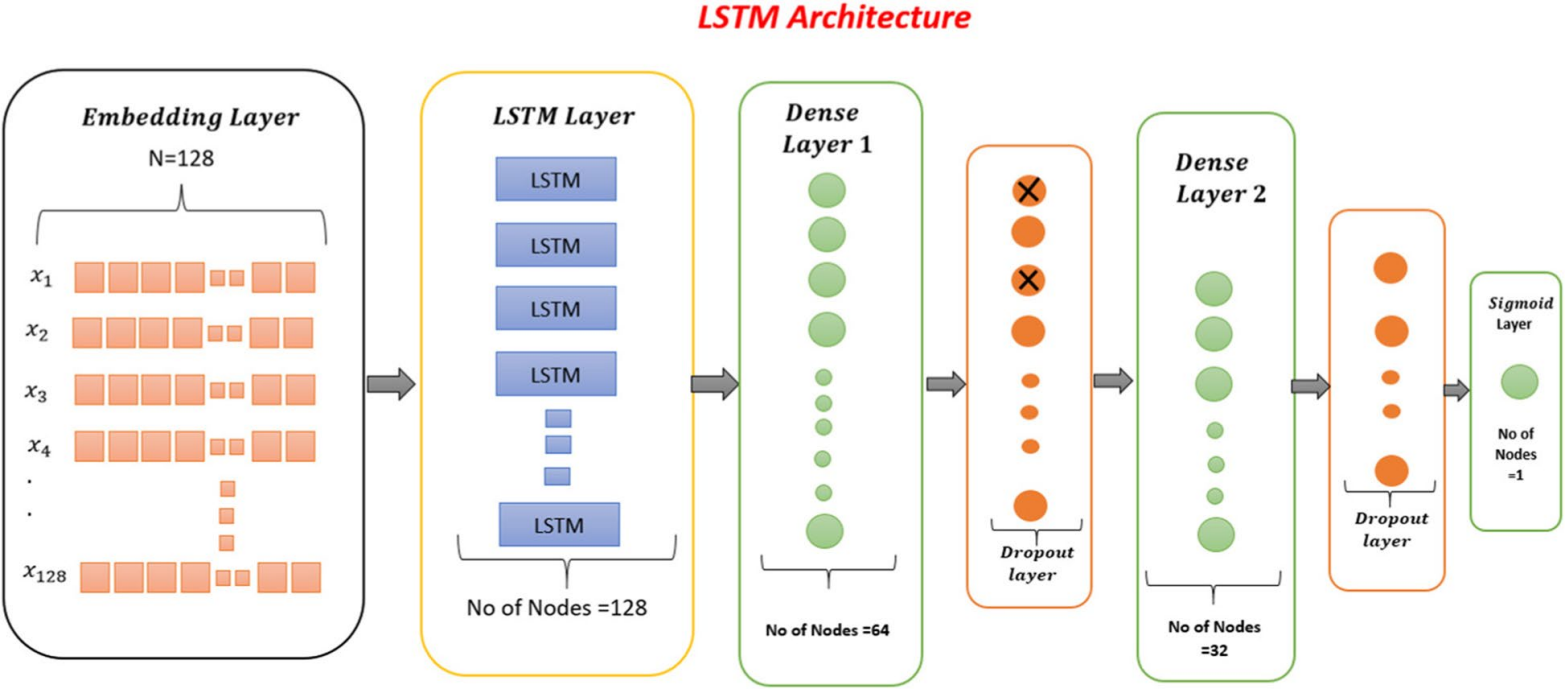
LSTM model used for identification of thyroid adenocarcinoma

Figure 2 shows that for each iteration of LSTM, there are 128 neurons in the embedding layer. These neurons are passed to the LSTM layer with 128 nodes. After the LSTM layer, there is a dense layer with 64 nodes. All these neurons pass to the dense layer, where filters are applied. The dropout layer is used to turn off some nodes to avoid overfitting. In the LSTM model, two dense layers, two dropout layers, and one sigmoid output layer are developed.

#### Gated recurrent unit (GRU)

GRU is also a gated technology in deep learning. Unlike LSTM, GRU uses only the update and reset gates [30].

**Fig. 3:**
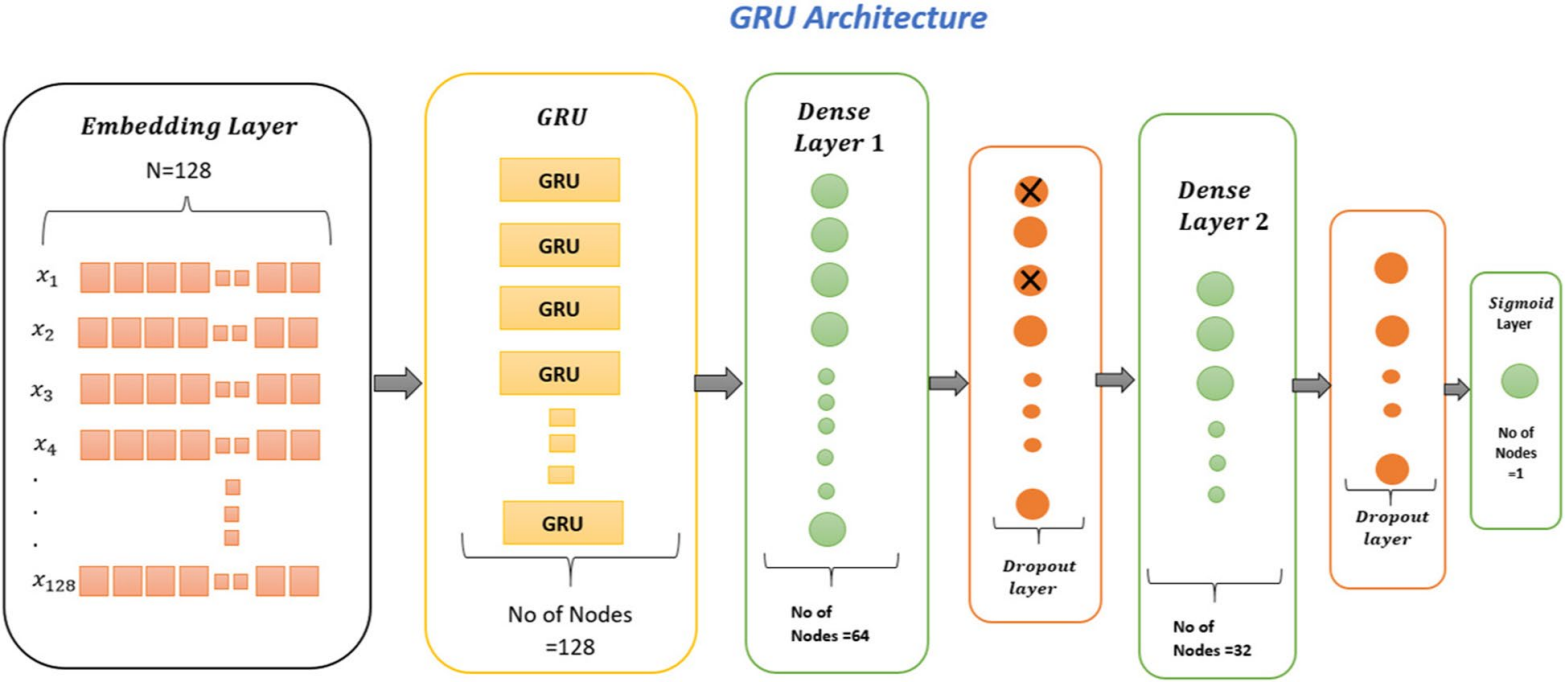
GRU model for identification of thyroid adenocarcinoma

Figure 3 explains that the proposed study used two dense layers, two dropout layers, and one output layer for GRU.

#### Bi-directional LSTM (BLSTM)

BLSTM works like LSTM but in both directions, backward and forward.

**Fig. 4:**
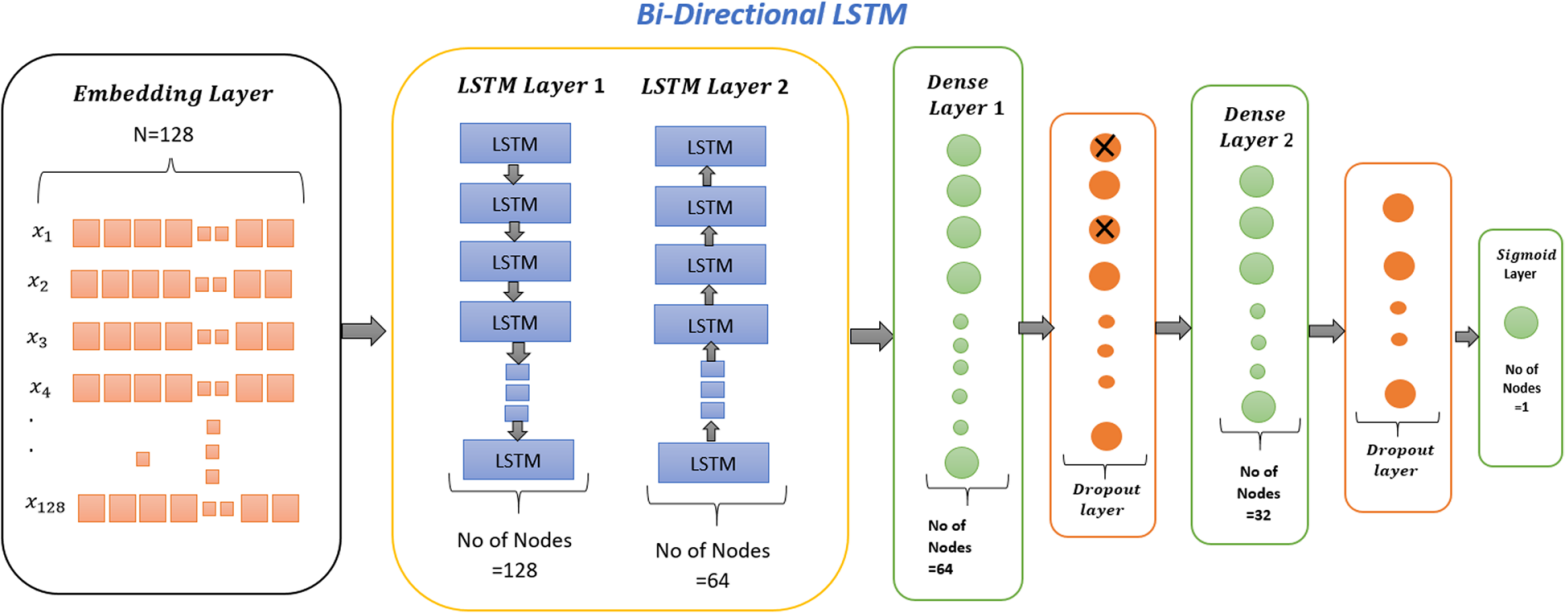
BLSTM cell structure for identification of thyroid adenocarcinoma

As Fig. 4 shows, two LSTM layers, forward and backward, are used in BLSTM, along with two dense layers, two dropout layers, and one sigmoid output layer.

#### Ensemble learning model (ELM)

There are many studies that utilize the deep learning techniques [49–53] but here in this study multiple deep learning models are ensemble. ELM combines multiple experts or classifier models in machine learning or deep learning to solve a specific computational problem [54]. It is one of the most widely used artificial intelligent approaches from the last two decades for improving predictive performance avoiding the overfitting of model, representation, and computational advantages. There are three main classes of data stream classification stacking, bagging, and boosting [55]. An ELM is developed by diversity, training the member classifier, and combining the classifier.

In the first step, the data samples are sampled from the database. Then, every instance is trained using ELM, and these instances are combined. For a given $\:k$ instance in a dataset, with feature $\:f$ the ELM is calculated by Eq. (14)

14 $$\acute{\text{y}} = \beta\:\left({x}_{i}\right)=G({f}_{1,}\:{f}_{2},\:{f}_{3}\dots\:.\:{f}_{k})$$

In Eq. (14), G represents the aggregate function with $\:{f}_{1,}{\:f}_{2}$ inducers for predicting the single output $\acute{\text{y}}$. $\:\beta\:$ is an ensemble learning model. The dataset is represented by.

15 $$D = \left\{\left({x}_{i},\:{y}_{i}\right)\right\}\left(\right|\text{D}| \: = \: \text{n},{x}_{i} \:\upepsilon\ \:\:{R}^{m},{y}_{i}\:\upepsilon \: \:\text{C})$$

Here $\:C,$ used for a Classification problem, $\:D$ is the data set with instances $\:{x}_{i}$ and $\:{y}_{i}$.

This study develops the proposed ELM by combining the identification efficiency of multiple deep learning models such as LSTM, GRU, and BLSTM.

The ELM is based on stacking method, which combines multiple base learners to improve overall performance. In stacking methodology in this study, several base models are trained, such as LSTM, GRU, BLSTM, on the training data. The predictions from these base models were then used as input features for a meta-learner, typically a logistic regression model, which learned how to best combine these predictions. This approach allows the meta-learner to identify and correct the weaknesses of the base learners, leading to improved accuracy and robustness.

In addition to explaining our chosen method, it is essential to compare it with other common combination mechanisms to provide a comprehensive understanding. The voting method, for instance, makes the final prediction based on the majority vote (for classification) or the average prediction (for regression) of the base models. While straightforward, this method may not capture complex relationships between the predictions. Another method, weighting, assigns different weights to the base models’ predictions based on their individual performance. Although more flexible than voting, it requires careful selection of the weights to be effective.

Stacking, on the other hand, trains a meta-learner on the base models’ predictions, enabling it to leverage the strengths of each base model more effectively. This method often outperforms both voting and weighting by learning how to best combine the predictions in a data-driven manner. We chose stacking due to its superior ability to model complex interactions between the base models’ predictions, leading to better overall performance.

### Statistical tools to evaluate the model

The model is trained on 300 epochs. For each model iteration, the accuracy increases, and the loss of the model decreases, as discussed in the result section. Multiple statistical tools are used to evaluate the proposed model, such as sensitivity, specificity, accuracy, F1 Score, precision, recall, loss, and AUC [56–59]. These are the most important evaluation measures used for binary classification. The mathematical equations of multiple statistical tools for model evaluation are explained in Eqs. (16, 17, 18, 19, 20, 21, 22, 23, 24)

16 $$\:\:Precision=\:\frac{TP}{TP+FP}$$

17 $$\:\:\:\:\:\:Recall\:\:\:=\:\frac{TP}{TP+FN}$$

18 $$\:F\:measure=\:\frac{2\left(Precision*Recall\right)}{Precision+Recall}$$

19 $$\:Cohe{n}^{{\prime\:}}s\:Kappa=\:\frac{{P}_{o}-{P}_{e}}{1-{P}_{e}}$$

20 $$\:Specificity=\frac{TN}{TN+FP}$$

21 $$\:Sensitivity=\frac{TP}{FN+TP}$$

22 $$\:Accuracy=\frac{TP+TN}{TP+FP+TN+FN}$$

23 $$\:MCC=\:\frac{\left(T\:P\:X\:T\:N\right)-\left(F\:P\:X\:F\:N\right)}{\sqrt{(T\:P+F\:P)(T\:P+F\:N)(T\:N+F\:P)(T\:N+F\:N)}\:}$$

24 $$\:AUC=\:\frac{P\left(\text{x}|\text{p}\text{o}\text{s}\text{i}\text{t}\text{i}\text{v}\text{e}\right)}{P\left(\text{x}|\text{n}\text{e}\text{g}\text{a}\text{t}\text{i}\text{v}\text{e}\right)}$$

The term accuracy correctly means identification of thyroid cancer and non-thyroid cancer. Precision refers to all the positively labeled as thyroid adenocarcinoma. Sensitivity and recall mean the number of positive class predictions. The F1 score is the average of recall and precision. Specificity refers to the identification of negatively labeled data. MCC refers to the difference between the actual and predicted values. Cohen’s kappa is used for classification accuracy.

## Results

The results of the SCT, IST, and 10-FCVT of ELM are presented in this study section.

### Self-consistency testing (SCT)

SCT is the first testing technique of the proposed model. The entire thyroid adenocarcinoma dataset is used for training and testing purposes with this testing technique. Table 3 explains the results of the SCT of the proposed ELM.

**Table 3:** Results of SCT of proposed ELM

Matrices	Values	Matrices	Values
Sensitivity	85%	Recall	86%
Specificity	87%	F1 Score	86%
Accuracy	86%	Precision	86%
MCC	0.73	Cohens Kappa	0.73

**Fig. 5:**
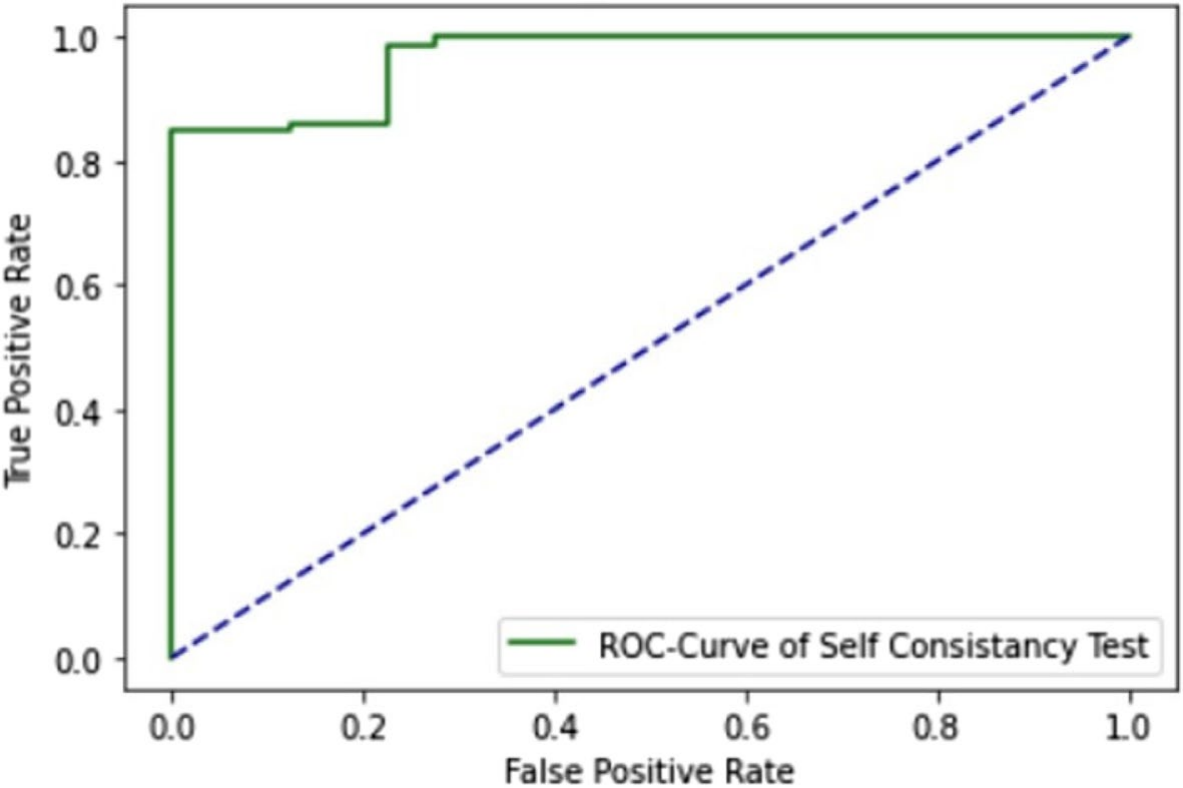
ROC curve of ELM using SCT

The ROC curve of SCT in ELM is explained in Fig. 5.

**Fig. 6:**
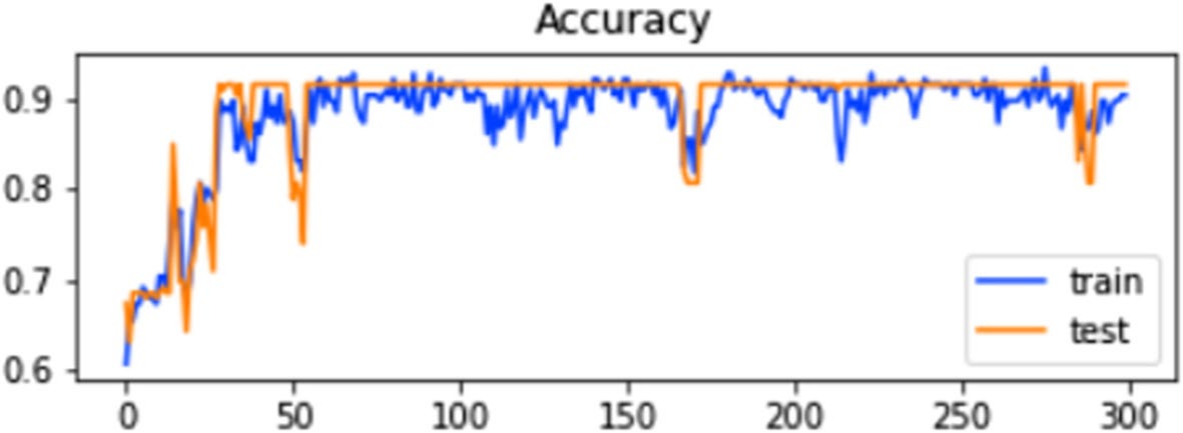
Training and testing accuracy of ELM in SCT

**Fig. 7:**
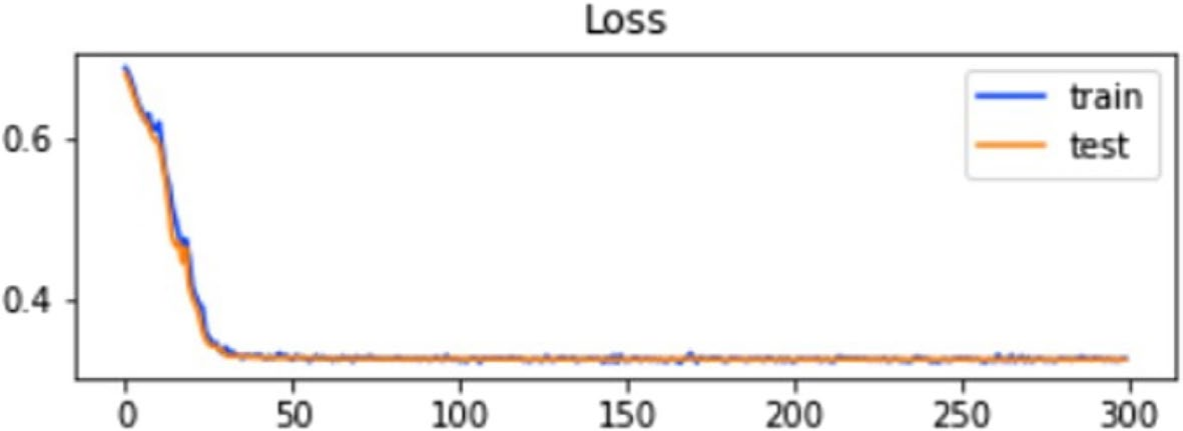
Training and testing loss of ELM using SCT

The ROC curve of the ELM in the SCT is presented in Fig. 5. Figure 5 illustrates how the model’s accuracy rises with each iteration of data. Both training and testing make use of the entire dataset. Figure 6 shows the model’s accuracy and Fig. 7 shows the loss graph during training and testing in the SCT.

### Independent set test (IST)

IST serves as the second testing method employed in the proposed research. The model’s accuracy is assessed through values extracted from the confusion matrix, which constitutes the primary performance evaluation technique. In this test, 20% of the dataset’s values are designated for testing, while the remaining 80% are utilized for model training. Table 4 provides a detailed overview of the IST results obtained for the proposed ELM.

**Table 4:** Results of ELM in IST

Matrices	Values	Matrices	Values
Sensitivity	92%	Recall	96%
Specificity	100%	F1 Score	96%
Accuracy	96%	Precision	96%
MCC	0.92	Cohens Kappa	0.92

**Fig. 8:**
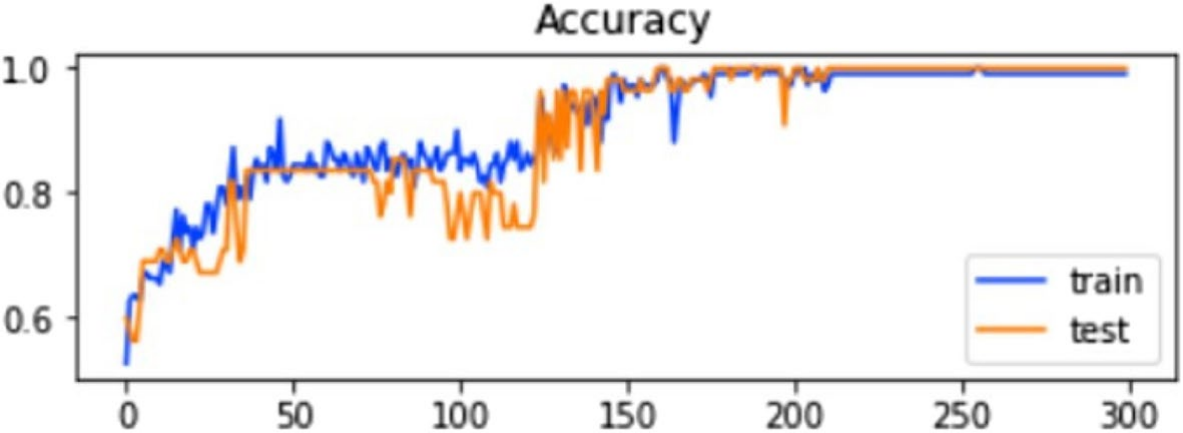
Training and testing accuracy of ELM in IST

**Fig. 9:**
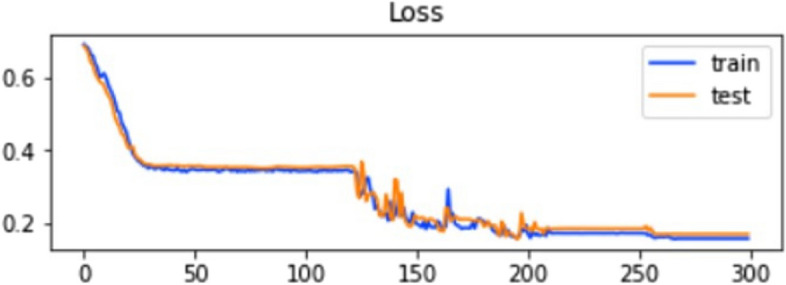
Training and testing loss of ELM in IST

The accuracy and loss of the training and testing dataset in IST are explained in Figs. 8 and 9.

**Fig. 10:**
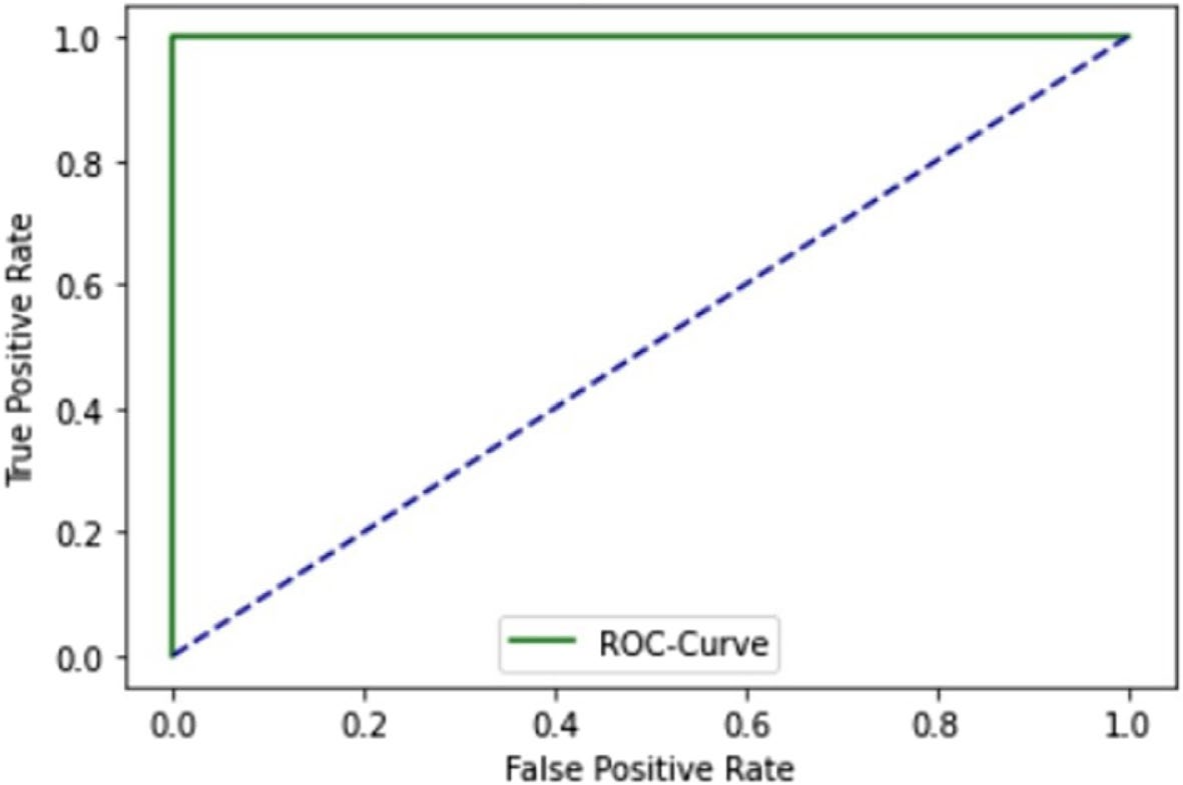
ROC curve of ELM in IST

The graphs show that the model’s accuracy increases rapidly with epochs, and at the same time, the value loss of the model goes downward. IST indicates the highest accuracy, 96%, from all three testing models. The ROC of the testing method is explained in Fig. 10.

### 10-Fold cross-validation test (10-FCVT)

10-FCVT stands as a prevalent testing method for machine learning algorithms. This approach involves partitioning the data into ten randomly selected segments. Subsequently, nine of these segments are allocated for training the model, while the remaining one serves for testing its performance. Table 5 shows the results obtained with 10-FCVT.

**Table 5:** Results of 10-FCVT of ELM

Matrices	Values	Matrices	Values
Specificity	88%	AUC	0.86
Sensitivity	85%	MCC	0.73
Accuracy	86%		

**Fig. 11:**
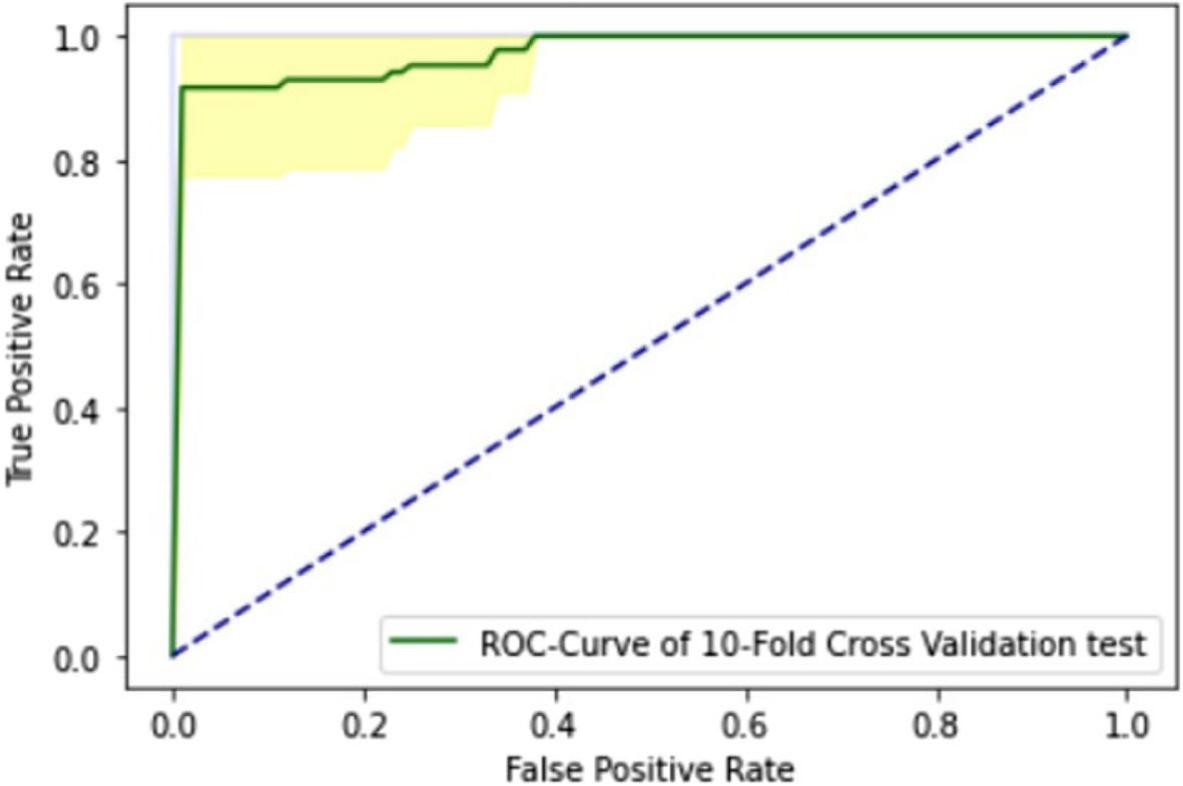
ROC curve of ELM in 10-FCVT

The ROC curve of 10-FCVT is explained in Fig. 11.

## Analysis and discussion

This study is proposed for the identification of thyroid adenocarcinoma, one of the most common cancers worldwide. This study is inspired by the ELM approach using LSTM, GRU, and BLSTM algorithms. Three testing techniques, including SCT, IST, and 10-FCVT, are used on these algorithms. The result of the testing is obtained in the form of sensitivity, specificity, accuracy, precision, recall, Mathew’s correlation coefficient, loss, F1 Score, training accuracy, and Cohen’s kappa. The combined results of these algorithms are explained in Table 6.

**Table 6:** Comparison of SCT, IST, and 10-FCVT of ELM

Evaluation Matrices	IST	SCT	10-FCVT
Accuracy (%)	96	86	86
Sensitivity (%)	92	85	85
Specificity (%)	100	87	88
MCC	0.92	0.73	0.73
Precision (%)	96	96	96
Recall (%)	96	96	96
F1 Score (%)	96	96	96
Cohens Kappa	0.92	0.92	0.92

Table 6 shows that the best accuracy is 96% for ELM in SCT, IST, and 10-FCVT, which offers almost the same results for ELM, 86%. IST also shows the highest sensitivity, specificity, and MCC value among all testing techniques. All three testing techniques give the same value of precision, recall, F1 score, and Cohen’s kappa for ELM.

This study achieves the highest accuracy in identifying thyroid adenocarcinoma compared to all previously introduced systems, as detailed in Table 1. Prior research had reported the highest accuracy of 94.6% for thyroid adenocarcinoma identification using machine learning algorithms such as Fisher’s discriminant ratio, Kruskal-Wallis’ analysis, and Relief-F on the SEER database [6]. However, the proposed model surpasses this accuracy, reaching 96%, even when compared to the latest models with larger datasets. Notably, the proposed model outperforms the Thynet model [20], designed for thyroid cancer diagnosis, which attained an accuracy of 92.2% using 18,049 images from 8,339 patients. The proposed ELM utilizes a dataset comprising 40 genes with 696 mutations across 633 samples, including both mutated and normal gene sequences.

The ELM achieved the highest accuracy of 96% with IST, significantly outperforming both SCT and 10-FCVT, each recording an accuracy of 86%. This indicates that the model performs exceptionally well when tested on an independent dataset, suggesting a high level of generalizability. In terms of sensitivity, IST again shows superior performance with 92%, compared to 85% for both SCT and 10-FCVT. This suggests that the ELM model is more capable of correctly identifying positive instances when evaluated independently than through sequential or 10-fold cross-validation methods.

The specificity results are particularly noteworthy, with IST achieving a perfect score of 100%, while SCT and 10-FCVT show closely matched performances at 87% and 88%, respectively. This perfect specificity under IST implies that the model has an excellent ability to correctly identify negative instances without any false positives in this validation context. The Matthews Correlation Coefficient, a balanced measure that takes into account true and false positives and negatives, also favors IST with a score of 0.92, compared to 0.73 for both SCT and 10-FCVT. This further confirms the robustness of the ELM model when validated against an independent test set.

Interestingly, the metrics for Precision, Recall, and F1 Score are consistent across all three validation techniques, each yielding a perfect 96%. This uniformity suggests that regardless of the validation method, the ELM model maintains a high level of reliability in balancing precision and recall. Cohen’s Kappa, which measures inter-rater agreement, is consistently high at 0.92 across all validation techniques. This consistency indicates that the agreement between the observed accuracy and the expected accuracy (by chance) is very strong, reinforcing the reliability of the ELM model’s predictions.

The comparative analysis reveals that the ELM model exhibits varying performance across different validation techniques. The standout performance of IST suggests that the model is highly effective when deployed on completely unseen data, demonstrating excellent generalizability and robustness. The perfect specificity score under IST is particularly impressive, indicating that the model is exceptionally good at avoiding false positives in this context. However, the performance dip observed in SCT and 10-FCVT, particularly in accuracy, sensitivity, and specificity, suggests that the model’s performance might be more variable under different sample distributions encountered in cross-validation methods. This variability could be attributed to the inherent differences in how these validation methods partition the data, potentially exposing the model to a wider range of sample variations and interactions.

Despite these differences, the consistency in precision, recall, F1 score, and Cohen’s Kappa across all validation techniques underscores the ELM model’s overall reliability and balanced performance. These metrics indicate that the model is consistently capable of correctly identifying positive instances and maintaining agreement between observed and expected accuracies, regardless of the validation method used.

**Table 7:** Results Comparion of LSTM, GRU, BLSTM, ELM

	Self-Consistency Test				Independent Set Test				10-Fold Cross-Validation Test			
	Acc	Sen	Spe	MCC	Acc	Sen	Spe	MCC	Acc	Sen	Spe	MCC
**LSTM**	86%	83%	87%	0.72	92%	90%	98%	0.90	84%	85%	84%	0.76
**GRU**	85%	85%	86%	0.81	90%	91%	97%	0.92	84%	84%	89%	0.71
**BLSTM**	88%	87%	89%	0.77	92%	93%	100%	0.92	86%	85%	85%	0.72
**ELM**	86%	85%	87%	0.73	96%	92%	100%	0.92	86%	85%	88%	0.73

The comparative analysis of LSTM, GRU, BLSTM, and ELM models reveals distinct performance strengths across various testing scenarios. In the SCT, BLSTM achieves the highest accuracy 88% and specificity 89%, while GRU excels in sensitivity and MCC, indicating robust internal consistency. In the IST, ELM deliver superior accuracy 96%, sensitivity 92%, and MCC 0.92, with specificity 100, highlighting their strong generalization capabilities to unseen data as shown in Table 7. During 10-FCVT, ELM again lead in accuracy 86, but LSTM stands out with the highest MCC 0.76, suggesting better overall predictive balance. GRU shows notable specificity 89% in this test. Overall, ELM exhibit consistent excellence across most metrics, particularly in handling IST, whereas BLSTM, GRU and LSTM demonstrate particular strengths in sensitivity and predictive correlation, respectively. These results underscore the nuanced trade-offs between different model architectures depending on the evaluation criteria.

### Limitations

Despite the promising results, this study has several limitations that need to be addressed in future research. Firstly, the dataset, while substantial with 633 samples, may not capture the full variability of thyroid adenocarcinoma cases; a larger and more diverse dataset could improve the model’s generalizability. Secondly, the high accuracy achieved with IST raises concerns about potential overfitting, as indicated by the perfect specificity score, suggesting that the model may not generalize well to other datasets. Moreover, a comprehensive comparison with a wider range of state-of-the-art models is necessary to contextualize the ELM model’s performance fully. The complexity of integrating GRU, LSTM, and BLSTM algorithms into the ELM model also poses significant computational demands, suggesting a need for model simplification or optimization. Lastly, the study does not consider longitudinal data, which could provide more comprehensive insights into the disease’s progression and treatment. Addressing these limitations in future research could lead to the development of more robust, generalizable, and clinically applicable models for thyroid adenocarcinoma identification.

## Conclusions

This study is for the identification of one of the most common cancers, thyroid adenocarcinomas. As discussed in the literature review section, many studies have proposed detecting thyroid adenocarcinoma, but none used the ELM approach. The ELM proposed integrates three distinct deep learning architectures: GRU, LSTM, and BLSTM. It employs an extensive dataset comprising both normal and mutated gene sequences for training and testing purposes. Evaluation of the model is conducted using three testing techniques: SCT, IST, and 10-FCVT. All three testing methods show the AUC value of 1.0 for ELM, shown in Figs. 1 and 6, and 7. The model accuracy increases with each epoch, while the loss decreases with every epoch. The highest accuracy, 96%, is obtained from IST, the highest accuracy from all the models for identifying thyroid adenocarcinoma to date, as discussed in the literature review Table 1.

This study gives a maximum accuracy of 96% with a huge dataset. In the future, another deep learning model can be developed to improve the accuracy and generate a more generalized dataset.

### Future work

The promising results of this study in identifying thyroid adenocarcinoma using the ELM model inspire several avenues for future research. Building on the robust performance and high accuracy achieved, future work should focus on integrating additional data sources from various genomic databases and clinical records to improve model generalizability and robustness. Exploring advanced deep learning architectures, such as Transformers and convolutional neural networks (CNNs) tailored for genomic data, may yield better performance and new insights. Real-world clinical validation through trials in diverse healthcare settings will be crucial to confirm the model’s practical utility and effectiveness. Incorporating multi-omics data can offer a comprehensive view of the disease’s molecular mechanisms, while developing explainable AI models will enhance clinician trust and facilitate adoption in clinical practice. Optimizing computational efficiency will ensure scalability and real-time application, and expanding the ELM model framework to other cancer types can test its versatility and adaptability. More effective ensemble strategies and parameter tuning techniques will be adopted to enhance the performance of the proposed ensemble model in future iterations.

## Data Availability

Data collection and pre-processing is one of the key factors for training a model. Before feeding the data to the model, the essentials are cleaning, pre-processing, and normalizing [32]. The pre-processed dataset gives efficient machine learning and deep learning model results. There is no generalized dataset available for thyroid adenocarcinoma. So, the proposed study developed a complete process for data collection. The normal gene sequences for thyroid adenocarcinoma are extracted from asia.ensambl.org [28], and the mutated gene sequences for thyroid adenocarcinoma are extracted from intogen.org [27]. Web scrapping code [33] is written in Python to automatically extract the required normal gene sequences from a well-known database, asia.ensembl.org, and the associated mutation information from a well-known mutation database, intogen.org. Mutated sequences are obtained by applying mutation information on normal gene sequences. Driver mutation causes cancer [34, 35]; therefore, only driver mutations related to thyroid adenocarcinoma are considered while creating the mutated dataset. There are 696 gene mutations involved in thyroid adenocarcinoma caused by 40 driver genes. All the samples are collected from persons of different ages, genders, treatments, cancer, and normal physical conditions. 696 mutations are used to train, test, and validate the model. Table 6 shows 40 genes related to thyroid adenocarcinoma, having 696 mutations of 633 samples in the dataset.
